# Defining the need for a massive transfusion in severe blunt traumatic patients

**DOI:** 10.1186/cc12305

**Published:** 2013-03-19

**Authors:** T Umemura, H Ishikura, Y Nakamura, K Hoshino, T Nishida, T Kamitani

**Affiliations:** 1 Fukuoka University, Fukuoka, Japan

## Introduction

We do not have enough criteria to make a judgment of the need for a massive transfusion (MT) in severe blunt traumatic patients. As a scoring system to predict the need for a MT, we usually use the Assessment Blood Consumption score (ABCs) and/or the Trauma-Associated Severe Hemorrhage score (TASHs). However, for these scoring systems, the procedure is slightly complicated. The aim of this study was to establish a predictor of a MT using coagulation or fibrinolysis markers.

## Methods

A retrospective analysis of MT was conducted in patients with severe blunt traumatic injury, which was defined as Injury Severity Score (ISS) of 16 or more admitted to the ICU between 1 June 2009 and 31 December 2010. Blood samples were collected from patients immediately after arriving at our level I trauma center. We defined the patients who received more than 10 unit packed red blood cells (PRBCs) within the first 24 hours as a MT group and who received less than 9 units PRBCs as a non-MT group. After the demographic data, number of units of PRBCs and the need for massive transfusions were recorded and analyzed in each groups, we compared data between two groups.

## Results

There were 114 patients who met the inclusion criteria. Fifty patients received blood transfusions (43.9%; 50/114). There were 27 patients in the MT group (23.7%; 27/114) and 87 in the non-MT group. The MT group was significantly higher in the ratio of females (*P <*0.001), ISS (*P <*0.01), PT-INR (*P <*0.001), APTT (*P <*0.05), ABCs (*P <*0.001) and TASHs (*P <*0.001) than in the on-MT group. On the other hand, the MT group was significantly lower in Ps (*P <*0.05) and fibrinogen level (*P <*0.001) than the non-MT group. In the receiver operating characteristics (ROC) analysis, the area under the curve (AUC) to distinguish a MT was the highest for TASHs (0.831, *P <*0.001), followed by fibrinogen (0.758, *P <*0.001), and ABCs (0.732, *P <*0.001). Fibrinogen was only a predictor of a MT without a scoring system such as ABCs and TASHs, and the optimal cutoff value was 205 mg/dl.

## Conclusion

We found that the level of fibrinogen was the most valuable predictor of a MT in the coagulation or fibrinolysis markers. It is certain that the level of fibrinogen at admission was not as useful as the TASHs about predicting a MT in this study. Whereas the scoring systems require the assessment of several factors, the measurement of fibrinogen is simple, easy and quick. We strongly suggest that the level of fibrinogen will be a useful predictor of a MT at in severe blunt traumatic patients.
